# Precise two-dimensional D-bar reconstructions of human chest and phantom tank via sinc-convolution algorithm

**DOI:** 10.1186/1475-925X-11-34

**Published:** 2012-06-20

**Authors:** Mahdi Abbasi, Ahmad-Reza Naghsh-Nilchi

**Affiliations:** 1 Department of Computer Engineering, Engineering Faculty, University of Isfahan, Isfahan, Iran

**Keywords:** EIT, D-bar, Sinc-convolution, Accuracy measures, Chest phantom, Human chest

## Abstract

**Background:**

Electrical Impedance Tomography (EIT) is used as a fast clinical imaging technique for monitoring the health of the human organs such as lungs, heart, brain and breast. Each practical EIT reconstruction algorithm should be efficient enough in terms of convergence rate, and accuracy. The main objective of this study is to investigate the feasibility of precise empirical conductivity imaging using a sinc-convolution algorithm in D-bar framework.

**Methods:**

At the first step, synthetic and experimental data were used to compute an intermediate object named scattering transform. Next, this object was used in a two-dimensional integral equation which was precisely and rapidly solved via sinc-convolution algorithm to find the square root of the conductivity for each pixel of image. For the purpose of comparison, multigrid and NOSER algorithms were implemented under a similar setting. Quality of reconstructions of synthetic models was tested against GREIT approved quality measures. To validate the simulation results, reconstructions of a phantom chest and a human lung were used.

**Results:**

Evaluation of synthetic reconstructions shows that the quality of sinc-convolution reconstructions is considerably better than that of each of its competitors in terms of amplitude response, position error, ringing, resolution and shape-deformation. In addition, the results confirm near-exponential and linear convergence rates for sinc-convolution and multigrid, respectively. Moreover, the least degree of relative errors and the most degree of truth were found in sinc-convolution reconstructions from experimental phantom data. Reconstructions of clinical lung data show that the related physiological effect is well recovered by sinc-convolution algorithm.

**Conclusions:**

Parametric evaluation demonstrates the efficiency of sinc-convolution to reconstruct accurate conductivity images from experimental data. Excellent results in phantom and clinical reconstructions using sinc-convolution support parametric assessment results and suggest the sinc-convolution to be used for precise clinical EIT applications.

## Background

Electrical impedance tomography is a new non-invasive imaging technique in which the conductivity distribution inside a body is reconstructed via knowledge of injected current patterns and resulted induced voltages through finite number of electrodes placed on its surface [1]. This modality has many medical applications including monitoring heart and lung functions [2,3], breast cancer detection [4] and diagnosis of pulmonary edema and diagnosis of the pulmonary embolus [5].

Reconstructing the conductivity images in EIT involves solving forward and inverse problems [1]. The solution of the forward problem is the potential distribution inside the body given the map of conductivity distribution. The inverse problem is to find the unknown conductivity map inside the body using finite sets of injected current patterns and measured voltages on the electrodes surrounding the body.

Algorithms for solving the forward problem of EIT use Finite Element Methods (FEM), Boundary Element Methods (BEM) and Finite Difference Methods (FDM)[1]. Existing approaches for solving the inverse problem of EIT include:

1. Linearized iterative methods such as Calderon’s method [6], back-projection [7,8] and NOSER [9], which are not able to reconstruct conductivity distributions with high variations [10].

2. Non-linear iterative methods such as equation error formulation [11], output least square [12], statistical inversion [13] and Newton–Raphson methods [14], which are accurate but suffer from the low convergence rate and high computational complexity [10].

3. Layer stripping methods [15] which are sensitive to noise and are weak in reconstruction of non-symmetric conductivities [10].

4. Direct algorithms such as D-bar [16] and Block method [17] which solve the full nonlinear inverse problem without any iteration in the conductivity domain and do not require any intermediate estimation of the conductivity from a forward model. Block method gains considerably from the homogeneity of conductivity distribution for particles inside each block of the body [17]. The problem of high computational burden faced in this method can be resolved by the method of modified equations [18]. Recently, a non-iterative linear inverse solution is introduced in [19] that raises the efficiency of this method via reduction in its computational complexity.

D-bar method is a new direct methodology, which was firstly introduced in the constructive proof of Nachman [16]. This method uses the properties of the D-bar operator of inverse scattering [20] to solve the full non-linear inverse conductivity problem on the planar domains with two degrees of derivatives. An overview of this method is provided in the following section. The reader can refer to [16] for more details. Note that, the quality of the reconstructed conductivity images by the D-bar method is highly affected by approximate numerical solution to a weakly singular integral equation, named D-bar [21-23].

Concerning the efficiency of the solution to D-bar equation, two different numerical methods, namely product integrals (PI) and multigrid (MG) are considered. PI-based methods to solve D-bar equation require *O*(*N*^6^) arithmetic computations on N-point grids which is huge even for advanced ultra-fast computers [21,23,24]. In addition, high error rates, reported in the reconstructed conductivity images of experimental phantoms using these methods [21] convinces the inefficiency of them for practical EIT.

The complexity and high rates of error of PI-based methods inspired the adaptation of MG methods [25] for solving D-bar integral equation. Although MG methods solve D-bar integral equation with a remarkable speed and decrease the computational burden from *O*(*N*^6^) to *O*(*N*^4^ log *N*) incorporating Fast Fourier Transform (FFT), the convergence rate of these methods may not reach ultra-linear levels [22]. Recently, Mueller [26] has employed MG solution of D-bar equation to reconstruct physical tank and human chest conductivity images. In addition to the presence of visual artifacts such as blurring, the position, size and orientation of the organs are not correctly reconstructed by MG.

These considerable drawbacks in aforementioned methods motivated us to present an effective computational algorithm based on sinc-convolution method to solve D-bar equation with higher accuracy and lower computational burden [27]. But, for an EIT algorithm to be practically used, some numerical and experimental proficiency tests are required to show its actual efficiency [10].

The aim of this study is to assess the feasibility of empirical conductivity image reconstruction via sinc-convolution algorithm in the D-bar framework of EIT. A regular EIT algorithm evaluation requires a standard test methodology which is followed by some experimental reconstructions. In this study, the approved parametric test methodology of [2] is used to evaluate sinc-convolution algorithm based on the reconstructions of a specific synthetic model. The employed scenario is described subsequently. After parametric evaluation of the sinc-convolution, two sets of boundary data are used to qualitatively asses the reconstructions of sinc-convolution. Indeed, these experiments validate the parametric evaluations and show real potency of the sinc-convolution for clinical EIT. For the purpose of comparison, two other algorithms including MG and NOSER are implemented.

The paper is organized as follows. In the immediately following section, steps of the D-bar algorithm of Nachman are reviewed. Next, the sinc–convolution algorithm for solving D-bar integral equation is described. After establishing synthetic models and explaining phantom and clinical measurements, computations of performance figures are described. The parametric evaluation results of sinc-convolution, MG and NOSER are followed by their experimental reconstructions of a phantom tank and a human lung data.

## Methods

The EIT problem on a two-dimensional simply connected region Ω is modeled by the generalized Laplace equation as

(1) $$\nabla .(\gamma (x)\nabla u(x))=0\text{,}\;x=(x_{1},x_{2})\in \Omega \text{,}$$

where *γ*(.) and *u*(.) represent the conductivity of the domain and the electric potential, respectively. The Dirichlet boundary condition

(2) $$u(x)=f(x)\text{,}\;x=(x_{1},x_{2})\in \partial \Omega \text{,}$$

represents the known voltage distribution, *f*, on the boundary of the Ω_,_ resulted from injecting a known current density, *g*, on the boundary that corresponds to Neumann boundary condition

(3) $$g(x)=\gamma (x)\frac{\partial u}{\partial \nu }\text{,}\;x=(x_{1},x_{2})\in \partial \Omega \text{.}$$

Here, *v* denotes the outward normal on the boundary ∂Ω. The voltage-to-current map takes the given voltage distribution *f* on the boundary to current density distribution *g*. This mapping is also called Dirichlet-to-Neumann mapping and is denoted by Λγ in EIT literature [10].

Actually, the inverse conductivity problem as stated firstly by Calderon [6] is to uniquely determine the unknown conductivity distribution *γ* from the knowledge of Λγ. There have been extensive efforts to find and prove the uniqueness of the solution to this problem including the work of Nachman [16], Brown-Uhlmann [28] and recently Astala [29] for two-dimensional inverse conductivity problem. All of these researches are based on the D-bar method of inverse scattering [30].

### Methods: D-bar method of EIT

The essence of the D-bar method of EIT is to transform the conductivity equation to Schrödinger equation and use the D-bar approach of inverse scattering to solve the resulting equation. For more details about the theory, the reader is referred to [16]. Here, we only review D-bar equations from the constructive proof of Nachman [16] for solving inverse conductivity problem on a simply connected two-dimensional region with two derivatives.

Change of the variable Ψ=*γ*^1/2^*μ* and q=Δ*γ*^1/2^/*γ*^1/2^ and assuming that *γ* is a constant *γ*_*best*_ in the neighborhood of the boundary transforms the conductivity equation (1) to Schrödinger equation in whole *R*^2^[16]

(4) $$(-\Delta +q)\Psi (x,k)=0\text{,}\;x\in R^{2}\text{.}$$

Note that, in the D-bar method a point x=(x_1_*x*_2_)∈Ω may be identified as a point x=x_1_+ix_2_ where i^2^=-1 in complex plane. Also the complex parameter k=k_1_+ik_2_∈*C* may be identified as a point k=(k_1_,k_2_)∈*R*^2^. Using the assumption that γ is a constant, *γ*_*best*_ in the neighborhood of the boundary or equivalently q=0 outside the boundary, leads to another Schrödinger equation [16]

(5) $$(-\Delta +q)\gamma^{1/2}(x)=0\text{,}\;x\in R^{2}\text{.}$$

The key idea behind the proof of Nachman is that since two equations (4), (5) have same compact potentials q, the unique solution of equation (4) can be used to find the unique solution to equation (5). That is *γ*^1/2^(*x*) = *ψ*(*x*, *k*), *for x* ∈ *R*^2^. The unique solution *ψ*(*x*, *k*) to equation (4) is called exponentially growing solution which was first introduced by Faddeev [31]. This solution is asymptotic to *e*^*ikx*^ for large |*x*| or large |*k*|. Defining the function [16]

(6) $$\mu (x,k)=e^{-ikx}\psi (x,k)\text{,}$$

which is asymptotic to 1 and considering aforementioned key idea in the Nachman’s proof [16], the conductivity *γ*(*x*) can be computed as

(7) $$\gamma (x)={\left(\lim_{k\to 0}\;\mu (x,k)\right)}^{2}\text{.}$$

In the constructive proof of Nachman, an intermediate none-physical function named scattering transform of *q*(*x*) is defined as [16]

(8) $$t(k)=\int_{R^{2}}e^{i\bar{k}\bar{x}}q(x)\psi (x,k)dx\text{,}$$

which plays an important role in relating the measurement data and the conductivity distribution *γ*(*x*). Note that, in equation (8), *k¯* and *x¯* are respectively the complex conjugates of *k* and *x*. By simplifying the equation (8), the scattering transform is related to the Dirichlet-to-Neumann map using the formula [16]

(9) $$t(k)=\gamma_{\mathrm{best}}\int_{\partial \Omega }e^{i\bar{k}\bar{x}}\left(\Lambda_{\gamma }-\Lambda_{1}\right)\psi (.,k)d\sigma (x)\text{.}$$

Here, *Λ*_*γ*_ denotes the voltage-to-current density map when Ω has the conductivity distribution *γ* and *Λ*_1_ denotes the voltage-to-current density map for homogenous conductivity *γ* = 1. Using the large |*x*| asymptotic behavior *ψ*(*x*, *k*)|_∂*Ω*_ ≈ *e*^*ikx*^, an approximation to scattering transform of equation (9), namely *t*^exp^(*k*) is introduced [23] in the form

(10) $$t^{\exp}(k)=\gamma_{\mathrm{best}}\int_{\partial \Omega }e^{i\bar{k}\bar{x}}\left(\Lambda_{\gamma }-\Lambda_{1}\right)e^{\mathrm{ikx}}d\sigma (x)\text{.}$$

As shown in [32], as a regularization, the approximate computation of scattering transform *t*^exp^(*k*) should be restricted to a disk of radius *R* in the complex plane and should be set to zero outside the disk. Therefore, the approximate scattering transform *t*_*R*_^exp^(*k*)is defined as a compactly support function by [23]

(11) $$t_{R}{}^{\exp}(k)=\{\begin{array}{ll} \gamma_{\mathrm{best}}\int_{\partial \Omega }e^{i\bar{k}\bar{x}}\left(\Lambda_{\gamma }-\Lambda_{1}\right)e^{\mathrm{ikx}}d\sigma (x)\text{,}\; & \left|k\right|\leq R.\\ 0\text{,} & \left|k\right|>R \end{array}$$

The $t_{{}^{R}}^{\exp}(k)$approximation is used in some D-bar reconstructions using numerically simulated data [23,24,33], experimentally collected data on phantom tank [21] and human chest data [34].

It is shown by Nachman [16] that the connection between the scattering transform and the *μ*(*x*, *k*) is provided by D-bar equation as

(12) $${\bar{\partial }}_{k}\mu (x,k)=\frac{1}{4\pi \bar{k}}t_{R}^{\exp}(k)e_{-k}(x)\bar{\mu (x,k)}\text{,}$$

where $e_{-k}(x)=\exp(-i(xk+\bar{x}\bar{k})=\exp(-2i(x_{1}k_{1}+x_{2}k_{2})\text{.}$ This equation has a unique solution that satisfies two-dimensional singular D-bar integral equation [16]

(13) $$\mu (x,s)=1+\frac{1}{4\pi }\int_{R^{2}}\frac{t_{R}^{\exp}(k)}{(s-k)\bar{k}}e_{-k}(x)\bar{\mu (x,k)}dk\text{.}$$

In [27] a novel sinc-convolution algorithm is introduced for solving D-bar equation of (13). This sinc-convolution algorithm is based on using collocation to replace two-dimensional D-bar convolution equation by a system of algebraic equations. Separation of variables in the proposed method allows elimination of the formulation of huge full matrices and therefore reduces the computational complexity drastically. In addition, sinc-convolution method converges exponentially with a rate of $O(e^{-c\sqrt{N}})$. An overview of this algorithm is presented in the following. The reader is referred to [27] for more detail.

### Methods: numerical solution of D-bar equation via sinc-convolution

Here, the iterative sinc-convolution algorithm to solve the D-bar integral equation (13) is reviewed. The computational steps of sinc-convolution algorithm are enlisted in Table 1. As a matter of fact, the sinc-convolution method is used to replace the integral equation (13) by a system of algebraic equations.

**Table 1:** The sinc-convolution algorithm

***Step***	***Operation***
*1*	*Specify the bounds of D-bar integral equation*
	$\mu (x,k)=1+\frac{r(k)}{\pi }\text{,}\;\text{where}\;r(k)=\int_{-2R}^{2R}\int_{-2R}^{2R}\frac{t_{R}^{\exp}e_{-k}(x)}{4\bar{k}(s-k)}\bar{\mu (x,k)}dk_{1}dk_{2}\text{,}\;k=k_{1}+ik_{2}\in C,k\neq 0\text{.}$
*2*	*1. Decompose convolution integral *$r=\sum_{i=1}^{4}r_{i}$
	*2. Define mapping functions *$\varphi_{1}(z_{l})=\varphi_{2}(z_{l})=\varphi (z_{l})=\ln(\frac{z_{l}+2R}{2R-z_{l}})\text{,}\;l=-M,\ldots ,N\text{.}$
	*3. Compute sinc points *$z_{l}=\varphi^{-1}(lh)=\frac{2R(-1+e^{\mathrm{lh}})}{\left(1+e^{\mathrm{lh}}\right)},l=-M,\ldots ,N.$
	*4. Compute derivative of the mapping functions at sinc points *$\varphi '(z_{l})=\frac{4R_{}}{\left(2R+z_{l})(z_{l}-2R\right)}$
*3*	Use sinc matrices $I_{m_{i}}^{-1}(s,t)=\int_{0}^{s-t}\frac{\sin(\pi z)}{\pi z}dz+0.5\text{,}\;for\;s,t=-M,.,N\text{,}$
	$\begin{array}{l} A_{1}=hI_{m}^{-1}D(\frac{1}{\varphi '(z_{l})})=X_{1}S_{1}X_{1}^{-1},\\ A_{2}=h{\left[I_{m}^{-1}\right]}^{T}D(\frac{1}{\varphi '(z_{l})})=X_{2}S_{2}X_{2}^{-1}. \end{array}$
*4*	Compute the special “Laplace Transform” of the convolution kernel $g(k)=\frac{1}{k},k=k_{1}+ik_{2}\in C$G(u,v)=∫0∞∫0∞g(k1,k2)e−k1u+k2vdk1dk2=−vπ−π2(vu)+ln(vu)1+(vu)2+iuπ−π2(uv)+ln(uv)1+(uv)2,whereRe(uv)>0,Re(vu)>0.
*5*	**Iteratively**
	*1.* Compute each $r_{i}\;\text{,}\;i=1,.,4\text{,}$ using the separation-of-variables procedure of Table 2.
	*2.* Solve equation $\mu (k)=1+\frac{1}{\pi }(r_{1}+r_{2}+r_{3}+r_{4})$ to find *μ*(*k*).

Recall from the previous section that the support of scattering transform may be embedded in a disk of radius R. In the first step of the sinc-convolution algorithm the required bounds of two-dimensional convolution integral are determined as [ − 2*R*, 2*R*] × [ − 2*R*, 2*R*]. This provides the required knowledge to define the sinc-points via definition of region-related mapping functions in the next step of algorithm. In the second step of algorithm, the two-dimensional convolution integral in the right-hand-side of equation (13) is decomposed into four two-dimensional convolution integrals *r*_*i*_, *i* = 1, ., 4.

Third step of the sinc-convolution algorithm forms the required matrices for iterative solution of the D-bar equation. In the fourth step, a special “*Laplace transform*” of the kernel of the D-bar equation should be computed. This transform is used in the iterative computations of the sinc-convolution [27].

As clearly indicated in the fifth step of the sinc-convolution algorithm in Table 1, the separation-of-variables procedure of Table 2 is used to compute all four two-dimensional convolution integrals *r*_*i*_, *i* = 1, ., 4. This feature of the sinc-convolution allows computing a two-dimensional convolution integral *r*_*i*_, *i* = 1, ., 4, by only some one-dimensional vector operations.

**Table 2:** **Computing r**_**2 **_**using the separation of variables procedure**

***Step***	***Operation***
1	Form the array $\left[u_{i,j}\right]$
	$u_{i,j}=\frac{t_{R}^{\exp}(z)e_{-z}(x)}{4\bar{z}}\bar{\mu (z)}\text{,}\;\text{where}\;z=(z_{i},z_{j})for\;i,j=-M,\ldots ,N\text{.}$
2	Successively form the arrays
	$b_{.,j}^{+}=X_{{}_{1}}^{-1}u_{.,j}$
	$t_{i,.}^{+}=X_{{}_{2}}^{-1}b_{i,.}^{+}$
3	Form the products $t_{i,j}^{-}=G(s_{{}_{i}}^{1},s_{{}_{j}}^{2})t_{i,j}^{+}$
4	Successively form the arrays
	*b*_*i*,._^−^ = *X*_2_*t*_*i*,._^−^
	$r_{2_{i,j}}=X_{1}b_{.,j}^{-}$
	Note 1: $r_{2_{i,j}}$ are the approximations of *r*_2_ at *sinc* points (*z*_*i*_, *z*_*j*_).
	Note 2: In this procedure list, we used the notation *b*_.,*j*_ = (*b*_−*M*,*j*_, …, *b*_*N*,*j*_)^*T*^. Similar notations are used for *u*_.,*j*_ and *t*_*i*,._

Here, the algorithm for computing *r*_2_ is summarized and listed in Table 2. Note that, in the sinc-convolution method, as fully explained in [27], the separation of the variables of all four two-dimensional integrals in the D-bar equation may be done analogously.

Sum of these integrals reassembles the **r** matrix in the right-hand-side of the discrete-form D-bar equation as:

(14) $$\mu =1+\frac{1}{\pi }r\text{.}$$

Here, **μ** = *μ*_*ij*__*m*×*m*_ for *m* = *M* + *N* + 1 with elements *μ*_*ij*_ = *μ*(*z*_*i*_, *z*_*j*_). That is, the elements of this matrix are actually the values of the solution at *sinc* points. The **1**on the right hand side of the equation (14) denotes a vector of size *m*^2^ of 1’s. The equation (14) is solved by means of an iterative solver such as GMRES [35]. It is worth noting that since GMRES can only work with real-linear operators, the real and imaginary parts of the solution matrix, **μ**, must be kept separate [35].

### Methods: computational steps of D-bar reconstruction

**Figure 1:**
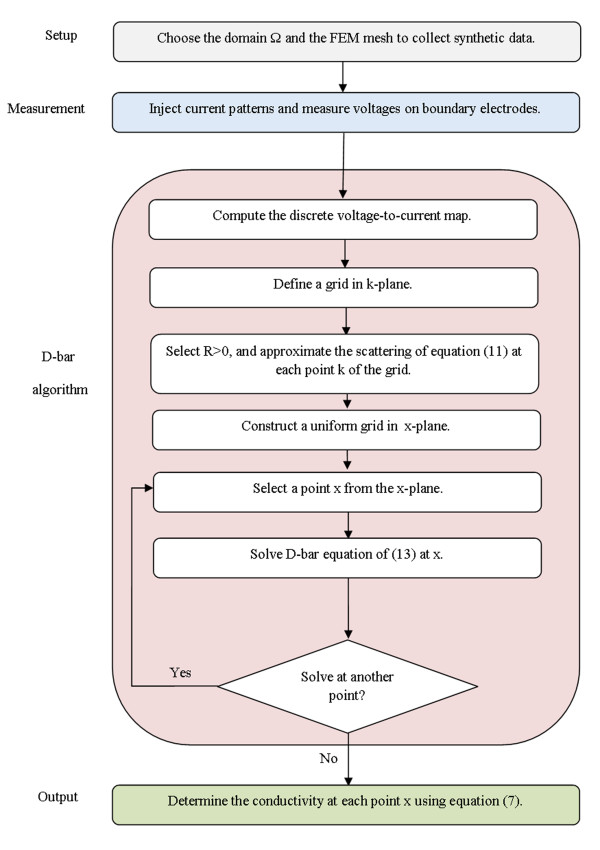
**The flowchart of the D-bar reconstruction algorithm.** Set up and measurement stages produce measurement data which is required for computing voltage-to-current map. The acquired mapping is used in D-bar algorithm to reconstructs the conductivity image.

To use both of the aforementioned datasets in the D-bar algorithm, the steps of the flowchart in Figure 1 must be followed. According to that flowchart, one may need to approximately compute the discrete form of the voltage-to-current map from the finite measurement data and then approximately compute the scattering transforms.

#### Computing the discrete dirichlet-to-Neumann map

In this study, known patterns of current are injected through the electrodes surrounding the body and the induced voltages on the same set of the electrodes are measured. Hence, the primary step in reconstruction is to construct the discrete version of the voltage-to-current density map in the form of a matrix from the injected current and measured voltage values. In this study, the method introduced by Isaacson in section 3 of [21] to construct the voltage-to-current density matrix from the boundary measurements on a phantom chest is followed. This computational method is used in all experimental D-bar reconstructions such as [26,34,36]. The reader is referred to [21] for analytical derivation of this approximation. Here, we briefly summarize that to fix the notations. Let

• L = the number of electrodes

• A = the area of an electrode, which is uniform in this study

•*Δθ* = the angle in radian between each electrode

• r = radius of the circular domain (in this study the radius of the tank).

In our study, L-1 trigonometric current patterns with amplitude M are used. The j-th current pattern on the *l-*th electrode is defined by [21]

(15) $$T_{l}^{j}=\{\begin{array}{cc} M\cos(j\theta_{l}), & j=1,\ldots ,\frac{L}{2}-1\\ M\cos(\pi l), & j=\frac{L}{2}\\ M\sin((j-\frac{L}{2})\theta_{l}), & j=\frac{L}{2}+1,\ldots ,L-1 \end{array}\text{.}$$

Let *t*_*l*_^*j*^ denote the vector of normalized currents $t^{j}=\frac{T^{j}}{∥T^{j}∥}$, where $∥T^{j}∥=\sqrt{\sum_{l=1}^{L}{\left(T_{l}^{j}\right)}^{2}}$. Also let *V*_*l*_^*j*^denote the voltage measured on the *l-*th electrode corresponding to *j-*th current pattern *T*^*j*^ and normalized so that $\sum_{l=1}^{L}V_{l}^{j}=0\text{,}\;j=1,\ldots ,L-1$. Then, the voltages v^*j*^ that would result from the normalized current patterns are given by $v^{j}=\frac{V^{j}}{∥T^{j}∥}$.

Let the (*u*(.), *w*(.))_*L*_ denote the discrete inner product defined by

(16) $${\left(u(.),w(.)\right)}_{L}=\sum_{1}^{L}\bar{u(\theta_{l})}w(\theta_{l})\text{.}$$

Then the entries of the discrete Neumann-to-Dirichlet map *R*_*γ*,*r*_ are approximated by [21]

(17) $$R_{\gamma ,r}(m,n)={\left(\frac{t_{l}^{m}}{A},v_{l}^{n}\right)}_{L}\text{,}\;where\;m,n=1,\ldots ,L-1.$$

Finally, by computing [21]

(18) $$L_{\gamma ,r}=R_{\gamma ,r}{}^{-1}\text{,}$$

one can obtain the discrete approximation of the Dirichlet-to-Neumann map *Λ*_*γ*_. Using the analytical method introduced in [21], the discrete current-to-voltage map *R*_1,*r*_ is approximated by the diagonal matrix

(19) $$R_{1,r}(m,n)=\frac{1}{A}\{\begin{array}{cc} \frac{1}{m}, & m=n\;and\;m,n\leq L/2.\\ \frac{1}{m-L/2}, & m=n\;and\;m,n>L/2\\ 0, & otherwise. \end{array}\text{.}$$

Similarly, the discrete approximation of the *Λ*_1_ is obtained by [21]

(20) $$L_{1,r}=R_{1,r}{}^{-1}\text{.}$$

Finally, computing [21]

(21) $$\delta L_{\gamma ,r}=L_{\gamma ,r}-L_{1,r}\text{,}$$

gives the discrete approximation to (*Λ*_*γ*_ − *Λ*_1_) .

#### Computing the scattering transform $t_{{}^{R}}^{\exp}(k)$

The series formulation for scattering transform$t_{{}^{R}}^{\exp}$, firstly derived by Isaacson in [21] and used in practical implementations of the D-bar including [21,26,34,36,37], is also used in this study. The reader is referred to [21] for analytical derivation and exact formulation of this approximate computation of the scattering transform. For each point z of the grid defined in k-plane, the approximated scattering transform is computed as [21]:

(22) $$\begin{array}{l} t_{{}^{R}}^{\exp}(z)\approx \gamma_{\mathrm{best}}\frac{Lr\Delta \theta }{2A}(\sum_{m=1}^{L/2-1}\sum_{n=1}^{L/2-1}a_{m}(\bar{z})a_{n}(z)\left(\delta L_{m,n}+\delta L_{m+\frac{L}{2},n+\frac{L}{2}}+i\left(\delta L_{m,n+\frac{L}{2}}-\delta L_{m+\frac{L}{2},n}\right)\right)\\ \;+\sqrt{2}(\sum_{n=1}^{L/2-1}a_{\frac{L}{2}}(\bar{z})a_{n}(z)\left(\delta L_{\frac{L}{2},n}+i\;\delta L_{\frac{L}{2},n+\frac{L}{2}}\right)\\ \;+\sqrt{2}(\sum_{m=1}^{L/2-1}a_{m}(\bar{z})a_{{}_{\frac{L}{2}}}(z)\left(\delta L_{m,\frac{L}{2}}-i\;\delta L_{\frac{L}{2}+m,\frac{L}{2}}\right)+2a_{\frac{L}{2}}(\bar{z})a_{\frac{L}{2}}(z)(\delta L_{\frac{1}{2},\frac{1}{2}}))\text{,} \end{array}$$

where

(23) $$a_{n}(z)=\{\begin{array}{cc} \frac{{\left(iz\right)}^{n}}{n!} & ,n\geq 0\\ 0 & n<0. \end{array}$$

The method of computing *γ*_*best*_, the best constant conductivity fit to measured data, is found in Appendix A.

#### Reconstruction of the conductivity

To reconstruct the conductivity *γ*(*x*) at each point *x* in the x-plane, first the D-bar equation of (13) is solved using the sinc-convolution method with different discretization levels in k-plane enlisted in the second column of Table 3 to find *μ*(*x*, *k*) and then the solution is evaluated to equation (7) at *k* = 0.

**Table 3:** Mesh/grid statistics used for forward models

**MODEL**	**Number of nodes**	**Number of elements**
**Thoracic region/Phantom tank/neonate chest**	790	1422
**Rotating circular target**	3281	6400

### Methods: models

Two sets of synthetic data, resulted from simulated experiments were used to parametrically evaluate the efficiency of the sinc-convolution based algorithm as well as other two methods. In addition, a dataset extracted from an EIT experiment on a phantom chest was used to validate the results of that assessment. Moreover, an EIT dataset measured on a human chest was used to illustrate the potency of the sinc-convolution in clinical applications. Note that, in all simulations and experimental reconstructions complete electrode model (CEM) [38,39] was used to represent the current density of electrodes. The meshing process was performed using NETGEN [40]. The type and number of mesh elements and nodes in forward and inverse solution of each simulation and experiment are enlisted in Tables 3 and 4, respectively. In each case, the forward problem mesh is finer than that used to solve the inverse problem. As a result, the forward problem is solved accurately; Meanwhile, this difference of meshes avoids the so-called “inverse crime” [10].

**Table 4:** Mesh/grid statistics used in inverse solutions

**MODEL**	**Mesh/Grid**	**Number of nodes**	**Number of elements**
Thoracic region/Phantom tank/Rotating circular target	Uniform grid	4096	3969
Neonate chest	Delaunay Mesh	8257	16256

#### Simulated models

##### Chest model

**Figure 2:**
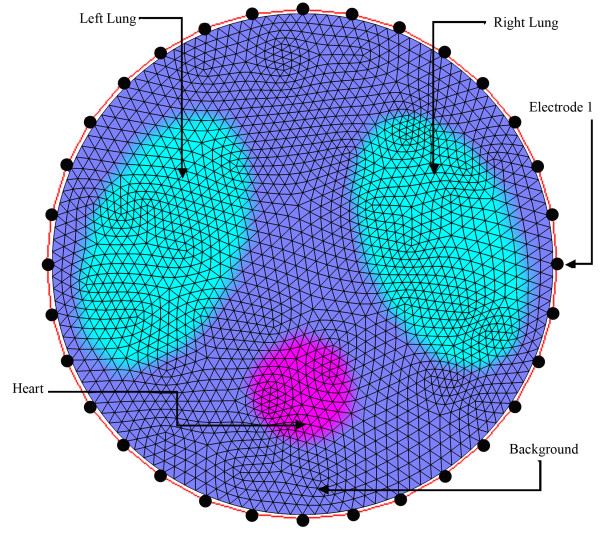
**The two-dimensional numerical model of thoracic region.** Elliptical regions are used to model the lungs and the circular region is used to model the heart. 32 equally spaced electrodes on the boundary inject current patterns and measure induced voltages.

A virtual chest phantom representing thoracic region of human body including two elliptical and one circular domain, respectively corresponding lungs and heart was used to evaluate the convergence rate of sinc-convolution, MG and NOSER. The second column of Table 5 includes the conductivity values of objects inside this numerically simulated chest phantom, as depicted in Figure 2.

**Table 5:** Conductivity values of organs inside chest phantoms

**Object**	**Conductivity of simulated chest (mS m**^**-1**^**)**	**Conductivity of experimental chest (mS m**^**-1**^**)**
Background	1000	424
Heart	1500	750
Lungs	500	240

As shown in Figure 2, data collection was simulated by 32 finite-sized boundary electrodes for current injection and voltage measurement like ACT3 system [42]. That is, 32 electrodes were arranged counter clockwise, with equally spaces on the boundary of a disk and the first electrode in the position of 3O’clock. The system could inject trigonometric current patterns [38,43] and measure voltages on all 32 electrodes simultaneously. The magnitudes of the injected current patterns were chosen to 1 mA. The simulated boundary values, along with the conductivities of the second column of Table 5, were used to solve the forward problem represented by equations (1) and (2) via FEM and as a result extract the boundary voltages.

##### Rotating circular target

A numerical model including a circular target with a diameter equal to 0.05 of the diameter of its container tank was used to evaluate the accuracy of sinc-convolution reconstructions via calculating some approved parameters. This model is introduced in [2] to evaluate the performance of EIT algorithms. In this model, the conductivity of the target is twice of the homogenous background conductivity.

Simulation data was generated from nine displacements of the target, starting from the medium center and progressing radially outward. The circular medium was surrounded by 16 electrodes. The amplitude of the injected current patterns was 1 mA. Simulated boundary values, were used to solve the forward problem represented by equation (1) and as a result extract the boundary voltages. In this study, to show the effect of measurement noise on the accuracy of under-test algorithms, a uniform noise with amplitude of 0.1 mA was added to resulted boundary data.

#### Experimental and clinical data

##### Chest phantom

**Figure 3:**
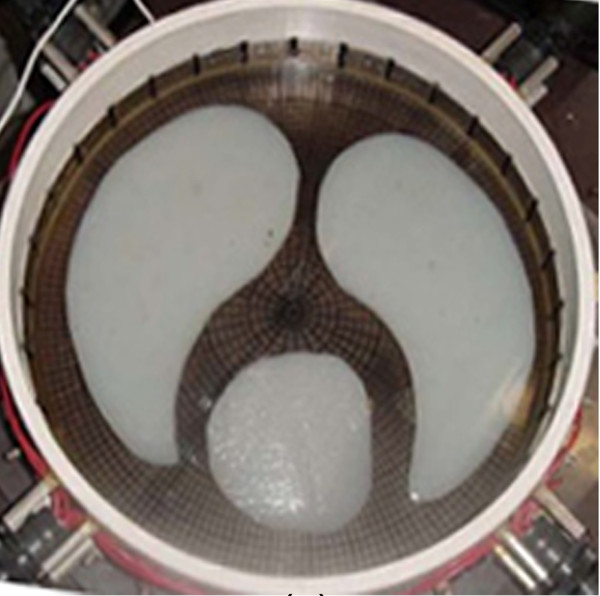
**The experimental chest phantom including agar heart and lungs in a saline tank [**[41]**].** Agar heart and lungs are suspended in a saline bath. 32 boundary electrodes inject current patterns and measure induced voltages on the boundary of the tank.

A boundary dataset extracted from real measurements was acquired from the EIDORS [41] website (http://eidors3d.sourceforge.net/data_contrib/jn_chest_phantom/jn_chest_phantom.shtml). The dataset is gathered by J. Newell, and D. Isaacson [21] in an experiment on a phantom chest consisting of agar heart and lungs in saline tank of radius 15 cm with 32 equally-spaced boundary electrodes of size 1.6 cm height and 2.5 cm width. Figure 3 shows the configuration of this experimental phantom. The conductivity values of the objects and the saline are included in the third column of Table 5.

##### Neonate chest data

A clinical EIT dataset collected by Heinrich et.al using Gottingen Goe-MF II device on a spontaneously breathing neonate [3] was found in EIDORS[44] website (http://eidors3d.sourceforge.net/data_contrib/if-neonate-spontaneous/index.shtml).

**Figure 4:**
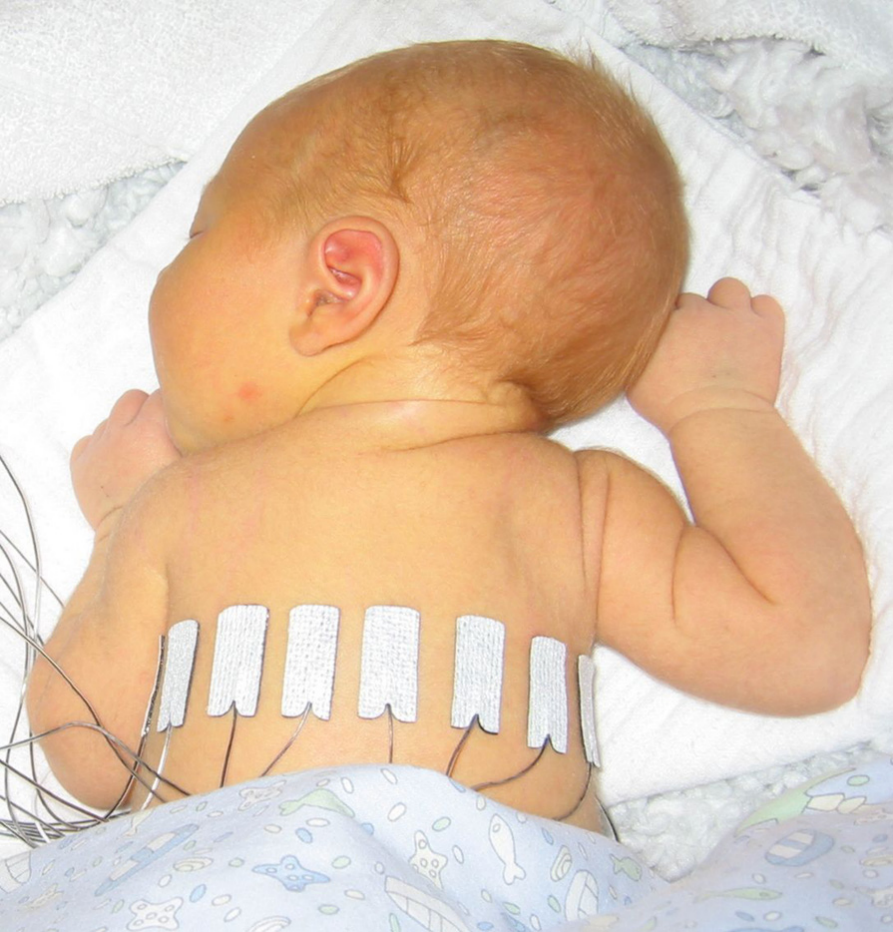
**The configuration of clinical EIT experiment on a neonate chest**[44]**.** The spontaneously breathing neonate is in prone position with the head turned to left. The first electrode is placed at the front of chest and electrodes 5, 9 and 13 are placed on left, back and right side of the chest respectively.

This data set includes 220 frames of measured voltages on 16 electrodes using adjacent protocol. As shown in Figure 4, in this measurement the neonate had been lying in prone position with the head turn to the left.

### Methods: Performance measures

#### Convergence rate versus grid size in k-plane

Convergence rate (CR) versus grid size in k-plane, is an important parameter showing the computational efficiency of EIT algorithms in D-bar framework. This calculation is motivated by [22] and calculated using reconstructions of synthetic thoracic region.

Let us denote the true conductivity as *γ*_*true*_ and denote the approximate solution with a grid of size *N*_*i*_, *i* = 1, …, 5 in k-plane as γ_*i*_. The supremum norm of the solution error may be defined as [22]:

(24) $$E_{i}=\sup\left|\gamma_{\mathrm{true}}-\gamma_{i}\right|\text{.}$$

Then, the convergence rate (CR) is defined as [22]:

(25) $$CR_{i}=\frac{E_{i}}{E_{i+1}}\text{.}$$

Note that, to compare sinc-convolution with other non D-bar algorithms such as NOSER, following performance measures are considered.

#### Accuracy measures versus target positions

Based on the approved test methodology introduced in [2], a scenario is arranged to parametrically evaluate sinc-convolution algorithm. As described below, in this scenario the reconstructions of the rotating circular target are used to calculate a set of accuracy measures that describe the quality of reconstruction algorithms.

Preliminarily, a one-fourth amplitude set γ_q_ is computed preliminarily based on reconstructions of circular target. This set contains all image pixels [γ]_*i*_, greater than one-fourth of the maximum amplitude:

(26) $${\left[{\text{γ}}_{\text{q}}\right]}_{i}=\{\begin{array}{c} 1\text{,}\;if\;{\left[\text{γ}\right]}_{i}\geq \frac{1}{4}\max(\text{γ})\\ 0\text{,}\;otherwise\text{.} \end{array}$$

A one-fourth threshold could guarantee to detect most of the visually significant effects in reconstructed conductivity images. The center of gravity of γ and γ_q_ are computed and the distances from the medium center to them are calculated as *r*_*t*_ and *r*_*q*_ respectively. Then the following performance measuring parameters are calculated.

• Amplitude response (AR) measures the ratio of image pixel amplitude in the target to that in the reconstructed image. For a circular target of area *A*_*t*_ with conductivity *σ*_*t*_in a medium with conductivity *σ*_*r*_[2]

(27) $$AR=\frac{\sum_{k}{\left[\gamma \right]}_{k}}{A_{t}(\frac{\sigma_{t}-\sigma_{r}}{\sigma_{r}})}$$

In this study, this parameter is normalized so that it AR = 1 for a circular target with $(\frac{\sigma_{t}}{\sigma_{r}})=2$ in the center of medium.

• Position error (PE) represents the extent to which reconstructed image truly represents the position of the circular target in the medium. This parameter is computed as [2]:

(28) $$PE=r_{t}-r_{q}\text{.}$$

• Ringing (RNG) measures the degree of opposite sign area surrounding the main reconstructed target area. For a circle *C* centered at center of gravity of γ_q_, the ringing could be obtained by [2]:

(29) $$RNG=\frac{A_{\mathrm{out}}}{A_{\mathrm{in}}}\text{.}$$

• Resolution (RES) is a measure of the smallest visible object within the reconstructed image. This parameter is be defined as [2]:

(30) $$RES=\sqrt{\frac{A_{q}}{A_{0}}}\text{,}$$

where *A*_*q*_ and *A*_0_ denote the number of pixels in γ_q_ and entire reconstructed image respectively.

• Shape deformation (SD) measure quantifies the fraction of γ_q_ which did not fit within a circle of an equal area. This parameter is computed as [2]:

(31) $$SD=\frac{\sum_{k\notin C}{\left[\gamma_{q}\right]}_{k}}{\sum_{k}{\left[\gamma_{q}\right]}_{k},}$$

where *C* denotes a circle centered at COG of γ_q_ with an area equivalent to *A*_*q*_.

## Results and discussion

All three methods were implemented within MATLAB and computations were performed in a Laptop with 2.4 GHZ CPU and 2 GB RAM. The methods were separately applied to the datasets extracted from aforementioned simulated and real models. To fairly compare the quality of reconstructed conductivity images, iteration parameters were set in a common range for all methods. In addition, same-size grids in k-plane were used in implementation of sinc-convolution and MG.

The following two steps were used to evaluate the quality of sinc-convolution images.

First, the synthetic reconstructions were evaluated via efficiency parameters of the preceding section. Next, reconstructions of physical and clinical models were used to validate the parametric assessments.

### Results of simulations

#### Convergence rate

The supremum of reconstruction errors and the required computation times for reconstructions of the synthetic chest phantom using MG and sinc-convolution with different levels of discretization in k-plane were measured according to equation (24) and then enlisted in third and fourth columns of Tables 6 and 7.

**Table 6:** Convergence rates and computation times of MG

**i**	**Discretization level in k-plane**	**E**_**i**_	**Solution Time (s)**	**CR**_**i**_
1	16	0.51309	61.12	1.82
2	32	0.28191	81.21	1.70
3	64	0.16582	152.33	2.03
4	128	0.08164	577.29	1.92
5	256	0.04252	3290.01	-

**Table 7:** Convergence rates and computation times of sinc-convolution

**i**	**Discretization level in k-plane**	**E**_**i**_	**Solution Time (s)**	**CR**_**i**_
1	16	0.44347	56.32	1.80
2	32	0.24637	65.27	3.51
3	64	0.07019	121.07	5.12
4	128	0.01370	339.93	9.84
5	256	0.00139	1871.32	-

Comparing corresponding accuracies of the reconstruction methods, one can notice that in each case the accuracy of the sinc-convolution method is much better than that of the MG, especially in reconstructions with large grids in k-plane.

Next, for each discretization level in Tables 6 and 7, the corresponding CR values were computed using the corresponding accuracies according to equation (25), and then enlisted in the fifth column of Tables 6 and 7. Comparing the corresponding convergence rates of the reconstruction algorithms shows that while the sinc-convolution method has a near-exponential convergence rate in reconstructing the conductivity distribution of the synthetic chest phantom, the MG method only converges with a linear rate, which is considered very slow. This result confirms the stated exponential convergence rate of sinc-convolution [45] as well as the linear convergence rate of MG [22].

Moreover, observing the computation times of sinc-convolution and MG in the fourth column of Tables 6 and 7, one may note that to obtain a low accuracy solution to the D-bar equation, the computational complexity of these two methods are approximately same, albeit, sinc-convolution method performs a fraction of time better than MG. However, to obtain a high accuracy solution, MG performs very poor. For example, while the sinc-convolution method converges to the approximate conductivity with accuracy of 10^-3^ in 1871 seconds, the MG can only achieve low accuracies not better than 10^-2^ in 3290 seconds, which is considered as a very poor performance. Now, it is predictable that to reconstruct higher resolution conductivity images in k-plane, the performance of the sinc-convolution would be finer than that of the MG.

#### Accuracy

**Figure 5:**
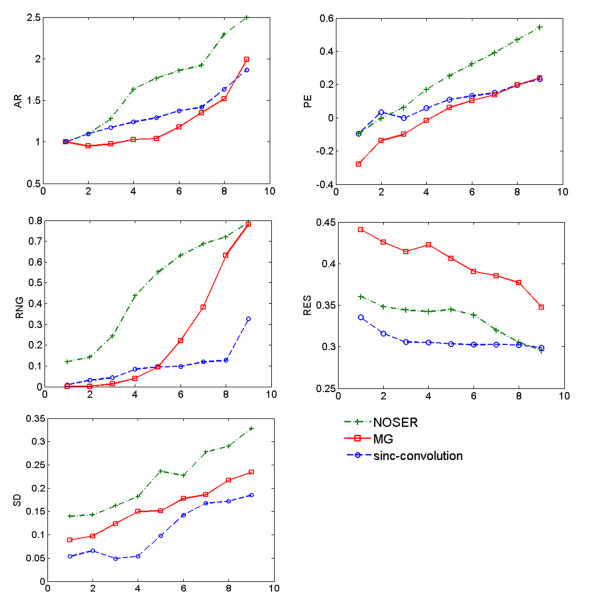
**The evaluation of the performance of algorithms using performance figures.** Plots correspond to AR, PE, RNG, RES, and SD of sinc-convolution, MG and NOSER.

The plots in Figures 5 illustrate different performance figures of each algorithm as functions of radial distance of the moving circular target from the medium center.

• The amplitude response of all three methods increase from the center of medium toward the boundary. Remarkable oscillations appear in the amplitude response of MG and NOSER respectively when the target is in the midway point and closest point to the boundary. Despite these two methods, the amplitude response of sinc-convolution is approximately uniform. This consistency guarantees that the same value of conductivities in different parts of the body contribute equally to the conductivity images produced by sinc-convolution.

• For position error, the plots show that when the target moves from the center to the boundary, the PE in MG, NOSER and sinc-convolution increases from −0.3, -0.1 and −0.1 to 0.2, 0.2, and 0.5 respectively. It is clear that the variance of PE in sinc-convolution curve is the closest one to zero. Therefore, the positions of objects are expected to be well recovered in the images reconstructed by sinc-convolution algorithm.

• The ringing plots indicate that for all three reconstruction algorithms, this artifact is increased as the target moves from the center of medium toward the boundary. The curves show that, for each position of the target, the maximum RNG is found in the image reconstructed by NOSER.

• Resolution plots show that the resolution of the NOSER and sinc-convolution are more uniform and considerably less than that of MG. It is clear that the RES of sinc-convolution is fractionally lower than NOSER. Therefore, one may expect to observe most of the conductivity details in sinc-convolution reconstructions.

• Shape deformation plots show that the SD of the target in sinc-convolution reconstructions is considerably less than that in images produced by each of other two algorithms. The optimum points for shape deformation in all three methods are near the boundary electrodes.

Aforementioned results evince the suitability of the sinc-convolution algorithm for experimental impedance imaging. In the following, reconstruction of experimental phantom tank via sinc-convolution is presented and compared with that of MG and NOSER.

### Results of experiments

#### Chest phantom

**Figure 6:**
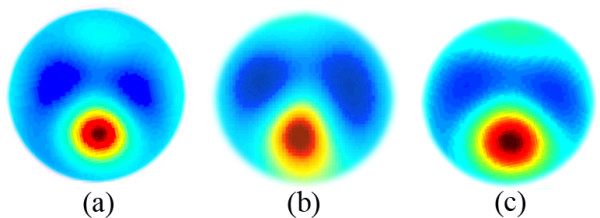
**The experimental reconstructions of chest phantom.** The resolutions of the images are 64 × 64. (**a**) The absolute reconstructed conductivity images using sinc-convolution*.* (**b**) The absolute reconstructed conductivity image using MG algorithm. (**c**) The absolute reconstructed conductivity image using NOSER algorithm.

Figure 6 illustrates reconstructions of the phantom tank using all three methods, derived on 64 × 64 grids in z-plane. Note that, this experimental model is reconstructed by product integrals (PI) method in [21] and MG method in [37].

The relative errors in reconstructing heart and lung, using under-test methods are enlisted in the second and third columns of Table 8, respectively. For the purpose of comparison, same parameters for the reconstruction results in [21,37] were computed and then enlisted in fourth and fifth rows of Table 8. It is clear that the relative errors in sinc-convolution reconstructions are the least.

**Table 8:** Maximum and minimum values of the chest phantom reconstructions

**Algorithm**	**Relative error of maximum**	**Relative error of minimum**	**Degree of truth**
MG	11	9	88%
NOSER	41	28	27%
Sinc-convolution	5	3	94.0
PI method [21]	12	23	93.1
MG with shape modeling [37]	7	7	93.0

Let define degree of truth (DT) of reconstructions as:

(32) $$DT=\frac{Max(\gamma_{\mathrm{rec}})-Min(\gamma_{\mathrm{rec}})}{Max(\gamma )-Min(\gamma )}\text{,}$$

where *γ*_*rec*_ and *γ* respectively denote the reconstructed and true conductivity. For each reconstruction experiment in the first column of Table 8, the corresponding DT is computed using equation (32) and then enlisted in the fourth column of Table 8. Comparing DT values show that the range of the conductivity distribution of the chest phantom is well recovered in sinc-convolution reconstruction.

It is clear that the representative results of this experiment in Figure 6, confirm the parametric results of Figure 5. The sinc-convolution reconstruction contains a number of sensible features, as described below.

• The overall size, position, and the orientation of the organs in the image produced by sinc-convolution are more accurate than that in Figures 6(c) and 6(d) produced by MG and NOSER.

• The sinc-convolution image recovers the separation between the two lungs well while MG and NOSER images do not; MG algorithm overestimates that distance and NOSER underestimates it.

• The distortion and blurring of the heart and lungs which are respectively evident in the MG and NOSER images are not appeared respectively in the sinc-convolution image.

• The degree of ringing artifact in sinc-convolution image of Figure 6(b) is less than that in MG image of Figure 6(c) and NOSER image of Figure 6(d)*.*

As can be seen, the representative results of this experiment agree very well with accuracy assessment plots in Figure 5. Therefore, the suitability of sinc-convolution for accurate phantom reconstructions is acknowledged.

#### Neonate chest

**Figure 7:**
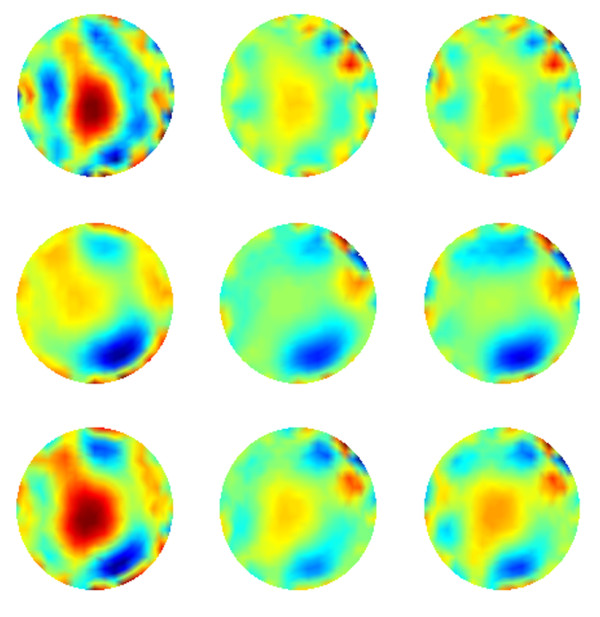
**The two –dimensional reconstructions of neonate chest.** First, second and third columns contain reconstructions of 45^th^, 70^th ^and 173^th ^frames of measured data. Top row: The reconstructed conductivity images using NOSER. Middle row: reconstructed conductivity image using MG algorithm. (c) Bottom row: reconstructed conductivity image using sin-convolution algorithm.

Two-dimensional conductivity images of the spontaneously breathing neonate chest are reconstructed using all three methods. The results are depicted in Figure 7. Note that, in these images anterior is at the top and right side of the neonate chest is reconstructed on the left side of the images. Images in the left, middle and right columns of Figure 7 correspond to 45th, 70th and 173th frames of data. These images illustrate the conductivity distribution of the neonate’s thoracic region in three end-inspirations.

It is worth noting that tidal volumes in the neonate’s left lung were reported less than those in his right lung [3]. That is, the conductivity of right lung is expected to be less than that of left one in reconstructed images. Comparing reconstructed images depicted in Figure 7, it is clear that this fact is well recovered in sinc-convolution results. In addition, the sinc-convolution reconstructions seem physiologically most accurate, demonstrating conductivity contrast of heart and lungs and recovering the approximate position of organs with least degree of ringing and deformation. It is evident that the reconstructions of other two methods are relatively distorted. One can easily notice an excellent agreement between numerical results obtained via parametric assessments and the quality of reconstructed images in Figure 7. As a result, the high degree of blurring in MG images may be caused by its low resolution and amplitude response. Similarly, the high degree of deformation of lungs and considerable ringing around them in NOSER images are previously predicted by SD and RNG curves of this method in Figure 5.

Note that, since exact information about the conductivity distribution inside the neonate’s chest is not available, no parametric evaluation and comparison could be planned. However, the representative results of this experiment and their correspondence to parametric evaluations confirm the feasibility of precise clinical EIT reconstruction using sinc-convolution.

## Conclusions

The feasibility of accurate practical conductivity image reconstruction via use of sinc-convolution algorithm in D-bar framework was investigated in this study. In the meantime, the performance of this algorithm was compared with two practical methods including, multigrid and NOSER. In this regard, a two-fold scenario was employed. In the first step, the quality of sinc-convolution reconstructions from noisy boundary data collected on specific synthetic models were evaluated against GREIT agreed accuracy parameters. Results show that the amplitude response and resolution of images are relatively better in sinc-convolution reconstructions. In addition, the effect of the distortions like position error, ringing and shape deformation is considerably reduced in the images produced by sinc-convolution method. Moreover, comparing the convergence rate of the sinc-convolution with that of MG shows that the new sinc-convolution method is computationally more efficient than its D-bar based competitor.

In the second step, conductivity images of an experimental phantom chest were reconstructed using all three methods. Excellent agreement between their qualities and parametric assessment results support the sinc-convolution suitability for experimental EIT. As a complementary experiment, two-dimensional conductivity images of the chest cross-section of a spontaneously breathing neonate were reconstructed using all three methods. A watchful comparison shows that the related physiological problem is best revealed in sinc-convolution images. In addition, position, size and orientation of organs are well recovered in sinc-convolution images.

These reasons, suggest the sinc-convolution as an efficient algorithm for precise clinical EIT applications.

## Appendix A: Computing *γ*_*best*_

The best constant conductivity approximation to the measured boundary data can be computed according to the following formula, which is found in [9,21].

Let *ρ* denote the resistivity (the reciprocal of the conductivity), then for a medium of homogenous resistivity, the voltage on the *l* th electrode from the *k*th current pattern is proportional to the voltage arising from a constant distribution of one. That is

(A.1) $$V_{l}^{k}(\rho )=\rho V_{l}^{k}(1)\text{.}$$

Let {*U*_*l*_^*k*^} denote the set of measured voltage data and *V*_*l*_^*k*^(*ρ*) the calculated voltage on the electrodes. To find the best constant resistivity fit to the data, one must solve the minimization problem

(A.2) $$\underset{\rho }{\text{min}}\sum_{k=1}^{L-1}\sum_{l=1}^{L}(\rho V_{l}^{k}(1)-U_{l}^{k})2.$$

The solution *ρ*_*best*_ to this minimization problem is given by

(A.3) $$\rho_{\text{best}}=\frac{\sum_{k=1}^{L-1}\sum_{l=1}^{L}(V_{l}^{k}(1)U_{l}^{k})}{\sum_{k=1}^{L-1}\sum_{l=1}^{L}(V_{l}^{k}(1))2}.$$

The best constant conductivity is then $\gamma_{\mathrm{best}}=\frac{1}{\rho_{\mathrm{best}}}$.

## Abbreviations

EIT: Electrical impedance tomography; PI: Product integral; MG: Multi-grid; CR: Convergence rate; DT: Degree of truth; AR: Amplitude response; PE: Position error; RES: Resolution; RNG: Ringing; SD: Shape deformation.

## Competing interests

The authors declare that they have no competing interests.

## Authors’ contributions

MA designed and performed the experiments and numerical modeling; ARNN analyzed the experiments and numerical modeling. Both of authors read and approved the final manuscript.
